# Dialogue between centrosomal entrance and exit scaffold pathways regulates mitotic commitment

**DOI:** 10.1083/jcb.201702172

**Published:** 2017-09-04

**Authors:** Kuan Yoow Chan, Marisa Alonso-Nuñez, Agnes Grallert, Kayoko Tanaka, Yvonne Connolly, Duncan L. Smith, Iain M. Hagan

**Affiliations:** 1 Cell Division Group, Cancer Research UK Manchester Institute, University of Manchester, Manchester, England, UK; 2 Biological Mass Spectrometry Facility, Cancer Research UK Manchester Institute, University of Manchester, Manchester, England, UK

## Abstract

The fission yeast scaffold molecule Sid4 anchors the septum initiation network to the spindle pole body (SPB, centrosome equivalent) to control mitotic exit events. A second SPB-associated scaffold, Cut12, promotes SPB-associated Cdk1–cyclin B to drive mitotic commitment. Signals emanating from each scaffold have been assumed to operate independently to promote two distinct outcomes. We now find that signals from Sid4 contribute to the Cut12 mitotic commitment switch. Specifically, phosphorylation of Sid4 by NIMA^Fin1^ reduces Sid4 affinity for its SPB anchor, Ppc89, while also enhancing Sid4’s affinity for casein kinase 1δ (CK1δ). The resulting phosphorylation of Sid4 by the newly docked CK1δ recruits Chk2^Cds1^ to Sid4. Chk2^Cds1^ then expels the Cdk1–cyclin B antagonistic phosphatase Flp1/Clp1 from the SPB. Flp1/Clp1 departure can then support mitotic commitment when Cdk1–cyclin B activation at the SPB is compromised by reduction of Cut12 function. Such integration of signals emanating from neighboring scaffolds shows how centrosomes/SPBs can integrate inputs from multiple pathways to control cell fate.

## Abstract (precis)

Events at the fission yeast equivalent of the centrosome, the spindle pole body, determine the timing of mitotic commitment and mitotic exit. Previous work has established that events on Cut12 drive commitment, whereas events on a distinct scaffold, Sid4 drive exit. Chan et al. now show how signaling on Sid4 influences commitment to explain the rationale for using the centrosome as a signaling center; centrosomal signaling supports integration of outputs from distinct inputs.

## Introduction

**Figure 1.**
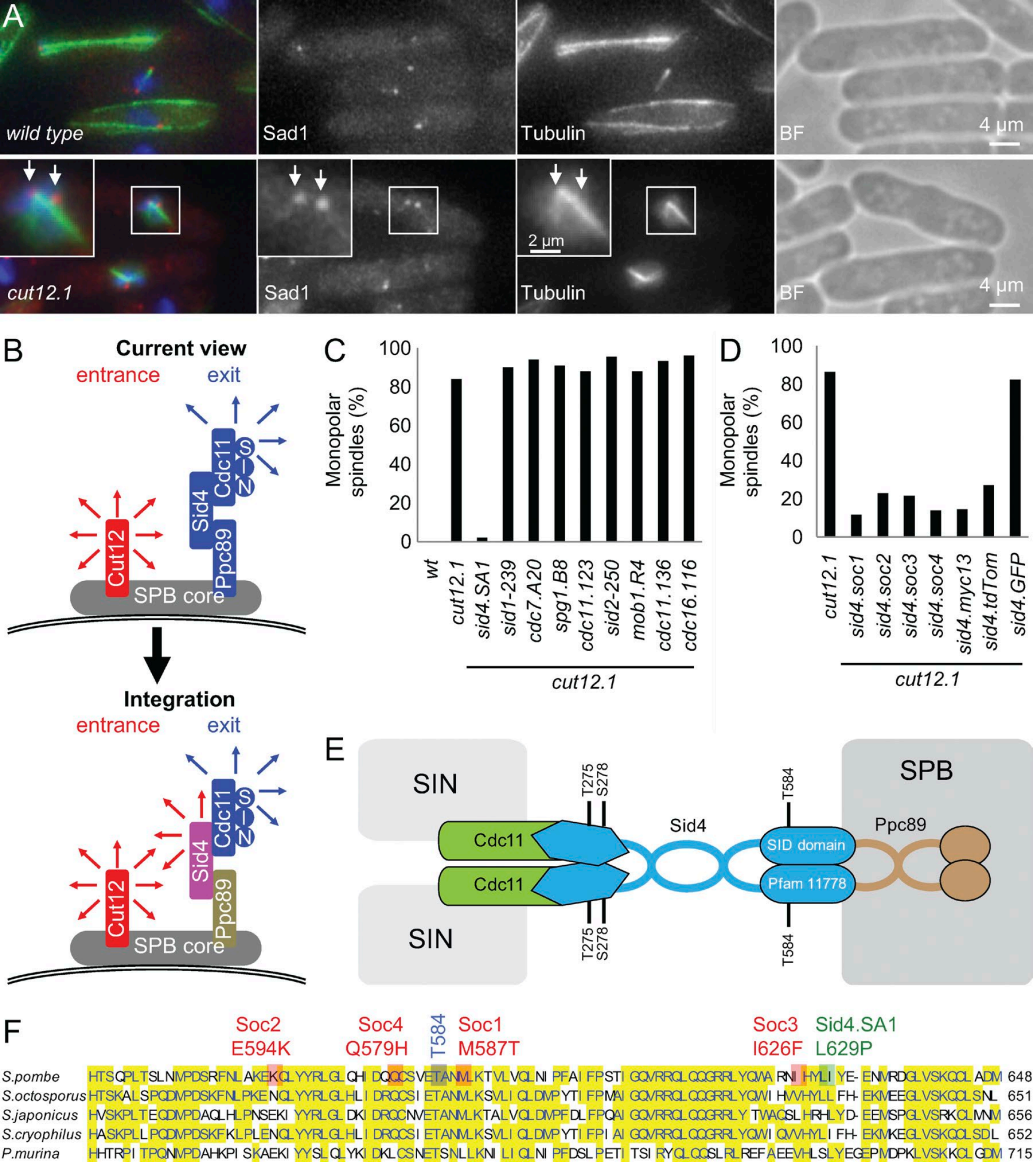
**C-terminal mutation of sid4 suppresses the *cut12.1* SPB activation defect.** (A) Representative images of immunofluorescence to reveal tubulin, the spindle pole marker Sad1, and chromatin 3 h after the temperature of an early log-phase culture was shifted from 25°C to 36°C in EMM2. *n* = 5. Arrows indicate the two SPBs. BF, brightfield. (B) A cartoon summarizing the SPB molecules upon which this study focuses. The representation is highly stylized because the mode of anchoring to the SPB core remains unclear for Ppc89 and Cut12, however. Cut12 is known to promote mitotic commitment (red arrows), whereas the anchorage of Sid4 to the SPB by Ppc89 enables Sid4 to anchor Cdc11 to the SPB. As Cdc11 recruits the SIN to the new SPB in anaphase, the recruitment of Cdc11 to Sid4 supports the events of mitotic exit such as septation and the formation of the equatorial microtubule-organizing center (Heitz et al., 2001; Simanis, 2015). (C and D) Representative graphs indicating the frequency of spindle monopolarity in samples of the indicated strains 3 h after early log phase cultures were shifted from 25°C to 36°C. For each strain, 100 cells with spindle staining were scored as being either bipolar or monopolar. *n* = 3. Note that the SPB activation delay of *cut12.1* means that monopolarity gives an underestimate of the incidence of SPB activation defects (Tallada et al., 2009). See also Fig. S1 A. (E) A schematic of the characterized associations of the indicated SPB components. The core SPB and SIN are indicated in gray. It is not clear whether only one or both of the components of the Sid4 dimer binds to Cdc11 or Ppc89. The coiled-coil regions in Ppc89 have prompted us to show Ppc89 as a dimer; however, we note that homodimerization or higher levels of oligomerization are yet to be demonstrated. (F) The position of key mutations within an alignment of the sequences of the C termini of Sid4 from *Schizosaccharomyces* species and *Pneumocystis murina*.

Cdk1–cyclin B activity is restrained through Wee1 kinase phosphorylation of Cdk1 until Cdc25 phosphatase removes this phosphate to promote mitosis. Cdk1–cyclin B activation then promotes polo kinase activity to further boost Cdc25 and inhibit Wee1 activity (Hagan and Grallert, 2013). In some systems, engagement of Polo feedback control influences the rate of mitotic commitment, whereas in others, Polo activity sets its timing (Kumagai and Dunphy, 1996; Lane and Nigg, 1996; Karaïskou et al., 1999; Karaiskou et al., 2004; Gavet and Pines, 2010; Aspinall et al., 2015). In fission yeast, the recruitment of protein phosphatase 1 (PP1) to the spindle pole body (SPB) component Cut12 sets the level of Polo^Plo1^ activity both locally on the SPB and globally throughout the cell (Mulvihill et al., 1999; MacIver et al., 2003; Grallert et al., 2013a,b). Blocking PP1 recruitment to Cut12 removes the requirement for Cdc25 (Grallert et al., 2013b). When Cut12 function is compromised by shifting the temperature-sensitive *cut12.1* mutant to the restrictive temperature of 36**°**C, Polo^Plo1^ activity falls, and the new SPB, which has been generated by conservative duplication, fails to nucleate microtubules, leading to cell cycle arrest with a monopolar spindle (Fig. 1 A; Bridge et al., 1998; MacIver et al., 2003; Tallada et al., 2009). The activation of this nonfunctional SPB by enhancement of Cdc25 levels shows that the inactivity of the new SPB arises from a failure to locally activate Cdk1–cyclin B on this SPB (Tallada et al., 2007, 2009). Thus, the *cut12.1* monopolar phenotype is a measure of the local role played by Cut12 in Cdk1 activation on the SPB (Fig. 1 B).

In addition to its role in driving the cell into mitosis, Polo^Plo1^ kinase is recruited to the SPB component Sid4 to control the timing of cytokinesis and septation by activating the septum initiation network (SIN; Fig. 1 B; Ohkura et al., 1995; Tanaka et al., 2001; Morrell et al., 2004). The SIN is a G protein–regulated network whose components and architecture are analogous to the core of metazoan hippo signaling and the budding yeast mitotic exit network (MEN; Simanis, 2015). Cnm67 and Sid4 are homologous MEN and SIN components that act in an identical fashion to anchor the budding and fission yeast networks to the SPB. The amino termini of Sid4/Cnm67 dock the orthologous SIN/MEN scaffolds Cdc11/Nud1, whereas the carboxyl termini anchor the complexes to their respective SPBs via the Pfam motif 11778 (Adams and Kilmartin, 1999; Elliott et al., 1999; Krapp et al., 2001; Schaerer et al., 2001; Tomlin et al., 2002; Morrell et al., 2004; Rosenberg et al., 2006; Klenchin et al., 2011). The anchor for Sid4 in the fission yeast SPB is Ppc89 (Fig. 1 B; Rosenberg et al., 2006). Recruitment of the RING finger ubiquitin ligase Dma1 to Sid4 delays septation when spindle function is perturbed (Murone and Simanis, 1996; Guertin et al., 2002; Johnson and Gould, 2011; Johnson et al., 2013).

To date, the mitotic entrance signaling events emanating from Cut12 have been assumed to operate independently of the signaling events on Sid4 (Fig. 1 B, top). However, we now find that a signaling relay on Sid4 promotes mitotic commitment. Thus, the decision to commit to mitosis integrates inputs from both Cut12 and Sid4 (Fig. 1 B, bottom). We discuss how the incorporation of the DNA replication checkpoint kinase Chk2^Cds1^ offers further potential for signals emanating from Sid4 to integrate inputs from replication/repair pathways into cell cycle control as reported for DNA checkpoint control by human centrosome components (Griffith et al., 2008; Barr et al., 2010).

## Results

### Sid4 mutations overcome the mitotic commitment defect of *cut12.1*

Because Sid4 recruits Polo^Plo1^ (Morrell et al., 2004), we combined the temperature-sensitive Sid4-inactivating *sid4.SA1* mutation (Balasubramanian et al., 1998) with the temperature-sensitive *cut12.1* mutation in an attempt to study the impact of compromising Cut12 function upon Plo1.GFP recruitment to the SPB in isolation of any influence of Sid4 upon Plo1.GFP recruitment. Unexpectedly, the *cut12.1 sid4.SA1* cells formed bipolar spindles to complete successive rounds of mitosis (Fig. 1 C). The inclusion of the *sid4.SA1* mutation had enabled the new SPBs to activate Cdk1–cyclin B. We therefore asked whether this *cut12.1* suppression was a specific feature of a Sid4 defect or whether deficiencies in other SIN components would similarly suppress the SPB activation defect of *cut12.1*; however, none did (Fig. 1 D). This independence from SIN function prompted us to isolate four *sid4.soc* (suppressor of *cut12.1*) mutations that suppressed *cut12.1* yet retained full SIN function (Figs. 1 D and S1 A). All *soc* mutations resided in the C-terminal septum initiation defective (SID) domain (Pfam 11778) that anchors Sid4 to the core SPB component Ppc89 (Fig. 1, E and F; Tomlin et al., 2002; Morrell et al., 2004; Rosenberg et al., 2006). *cut12.1* suppression by *sid4.13myc* and *sid4.TdTom* alleles in which the tag had been fused to the C terminus of Sid4 (Krapp et al., 2001; Grallert et al., 2006) further highlighted the ability of changes in the carboxyl terminal region of the mitotic exit anchor Sid4 to compensate for local Cdk1–cyclin B activation deficiency in mitotic commitment of *cut12.1* (Fig. 1 D). *sid4.GFP* did not suppress *cut12.1*.

### Sid4.T584E compromises SIN function and suppresses *cut12.1*

**Figure 2.**
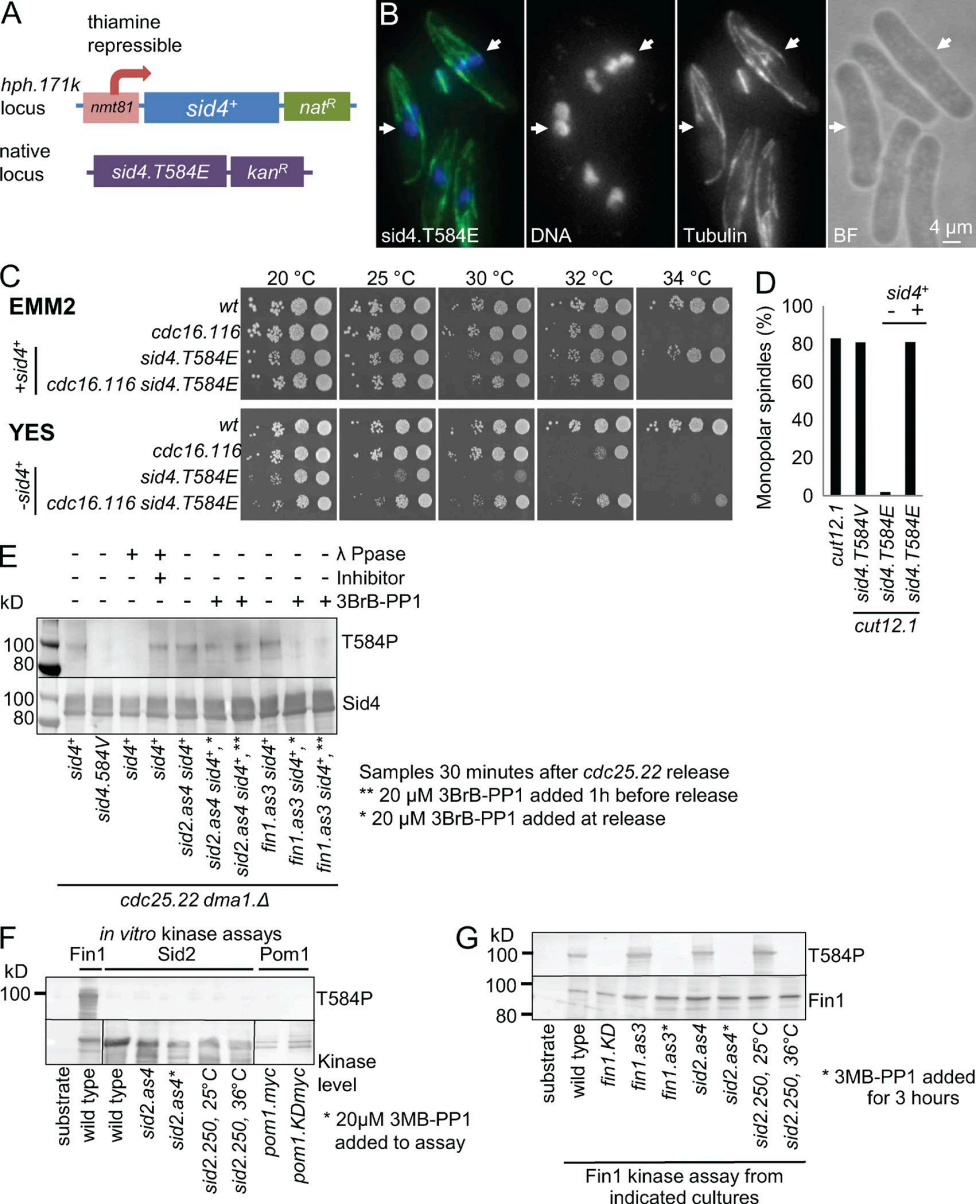
**Mutagenic mimicry of phosphorylation at T584 imparts a sin^−^ phenotype.** (A) A scheme showing the configuration of *sid4.T584E* strains. (B) Representative images of tubulin immunofluorescence as in Fig. 1 A of *sid4.T584E* cells 3 h after an early log phase culture was shifted from 19°C to 36°C. The binucleated interphase cells, indicated by arrows, arise from cytokinesis/septation failure in the previous cell cycle because of abolition of SIN function. *n* = 3. See also Fig. S2 B. BF, brightfield. (C) Spot tests of the indicated strains after growth on minimal (EMM2, no thiamine sid4^+^ expressed), or rich (YES) medium (contains thiamine to repress *sid4*^+^) at the indicated temperatures. (D) Representative monopolar counts as in Fig. 1 (C and D). See also Fig. S1 B. *n* = 3. (E–H) Western blots exploiting polyclonal antibodies that recognize Sid4 when phosphorylated on either T584 or simultaneously on both T275 and S278, all species of either Sid4 or Fin1, or the myc epitope tags on Pom1, as indicated. (E) Blots of Sid4 immunoprecipitates of denatured samples from *cdc25.22 dma1.Δ* cultures returned to 25°C 4.25 h after a shift to 36°C. As T584 phosphorylation peaks 40 min after release (not depicted), the impact of compromising kinase activities upon T584 phosphorylation was monitored at this time point in the figure. (F) Fin1 and Sid2 were isolated with polyclonal antibodies, whereas Pom1 was isolated with antibodies against the myc epitope, for kinase assays to identify which kinase could directly phosphorylate recombinant full-length Sid4 purified from *E. coli*. (G) Polyclonal antibodies precipitated Fin1 from asynchronous cultures of the indicated strains for in vitro kinase assays with recombinant Sid4. T584 phosphorylation was detected with the antibodies used in E.

Mapping sites of phosphorylation on Sid4 revealed phosphate on T584 (not depicted). The proximity of T584 to the *sid4.soc1* and *sid4.soc4* mutations (Fig. 1 F) prompted us to test the hypothesis that the *soc* mutations suppress *cut12.1* because they constitutively emulate a change in conformation/function that is normally transiently invoked by T584. If true, mutations that constitutively alter T584 phosphorylation should also suppress *cut12.1*. A repeated inability to generate the phosphomimetic *sid4.T584E* mutation at the native locus suggested that this mutation maybe lethal. We therefore modified the *sid4*^+^ locus to *sid4.T584E* in a host strain in which wild-type *sid4*^+^ was expressed from the *hph.171k* locus under the control of the thiamine-repressible *nmt81* promoter (Fig. 2 A; Basi et al., 1993; Fennessy et al., 2014). This strain was viable at all temperatures when *sid4*^+^ was expressed, yet was dead >20**°**C when thiamine had been added to repress *sid4*^+^ expression. Outcrossing the *nmt81:sid4*^+^ confirmed the presumed lethality of *sid4.T584E*, as colony formation failed at all temperatures (not depicted). The recessive temperature-sensitive phenotype of *sid4.T584E nmt81:sid4^+^* cells in the presence of transcription-repressing thiamine suggests either that the levels of wild-type Sid4 required to support *sid4.T584E* viability are extremely low or, we suspect, that *sid4.T584E* alone is unable to support the challenges of either spore viability or germination. All experiments with *sid4.T584E* therefore use *sid4.T584E nmt81:sid4*^+^ in the presence of thiamine.

*sid4.T584E* temperature-sensitive lethality is associated with the appearance of multinucleated cdc^−^ sin^−^ cells at temperatures >20**°**C (Fig. 2 B, arrows). This sin^−^ deficiency appeared to account for the lethality of *sid4.T584E* because enhancement of SIN signaling through inactivation of the SIN inhibitory component Cdc16 (Minet et al., 1979; Fankhauser et al., 1993) raised the *sid4.T584E* restrictive temperature from 25**°**C to 32**°**C (Fig. 2 C).

Having developed a strategy to study the *sid4.T584E* phenotypes, we found that *sid4.T584E* did indeed emulate the *sid4.soc* mutants in suppressing the SPB activation defect of *cut12.1* (Fig. 2 D). In contrast, *cut12.1* monopolar spindle counts were insensitive to incorporation of the *sid4.T584V* mutation to block phosphorylation (Figs. 2 D and S1 B).

### The NIMA kinase Fin1 phosphorylates T584

Antibodies that only recognize Sid4 when phosphorylated on T584 were generated to identify the kinase responsible for T584 phosphorylation (Fig. 2 E). Johnson and Gould (2010) have shown how the recruitment of the ubiquitin ligase Dma1 to Sid4 generates a complex ladder of Sid4-ubiquitin conjugates on Western blots that collapse to a simple smear of phosphorylated isoforms over the unphosphorylated protein upon deletion of *dma1*^+^. We therefore repeated their approach of incorporating a *dma1.Δ* deletion allele in all strains in which Sid4 phosphorylation was monitored by Western blotting in this study. Synchronization of mitotic progression through transient inhibition of Cdc25 revealed a peak in T584 phosphorylation just before septation (not depicted). We therefore used samples from this mitotic stage to monitor the impact of candidate kinases upon T584 phosphorylation. T584 phosphorylation was abolished by the addition of the ATP analogue 3BrPP1 to cells harboring an analogue-sensitive version of the NIMA-related kinase Fin1 (Fig. 2 E; Grallert et al., 2012). As the NDR kinase Sid2 activates NIMA^Fin1^ to control mitotic commitment in a Pom1-dependent manner (Grallert et al., 2012), either one of these three kinases could be the kinase that directly phosphorylates T584 of Sid4. We therefore used recombinant Sid4 in in vitro kinase assays of molecules isolated from yeast cells to show that NIMA^Fin1^ alone was able to directly phosphorylate T584 (Fig. 2 F). This ability of NIMA^Fin1^ to phosphorylate T584 relied on prior activation by Sid2 kinase in vivo before the kinase assay (Fig. 2 G).

### T584 phosphorylation reduces Sid4 affinity for the SPB anchor Ppc89 to invoke the SIN phenotype

**Figure 3.**
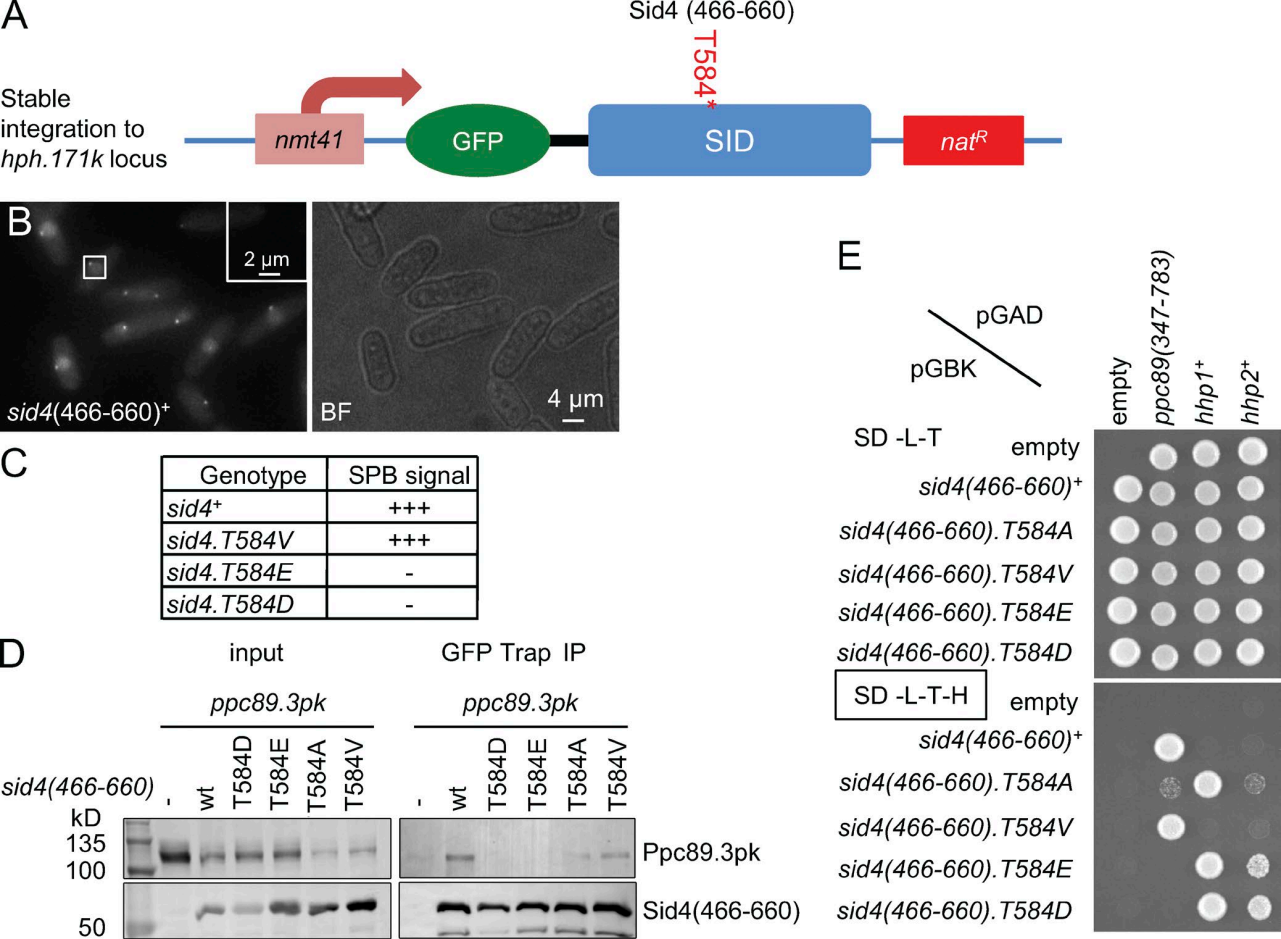
**T584 phosphorylation switches Sid4 affinity for Ppc89 to affinity for CK1δ^Hhp1^/CK1δ^Hhp2^.** (A) A scheme showing the configuration of strains expressing fusions between the carboxyl terminus of Sid4 and GFP to monitor affinity for the SPB in B. (B) Maximum projections of z stacks of GFP fluorescence that span the diameter of the cell. BF, brightfield. (C) Qualitative assessment of the impact of the indicated mutations upon the ability of the Sid4-GFP fusion protein to be recruited to the SPB. (D) GFP-Trap precipitation of the Sid4.GFP fusions used in A–C reveal association of wild-type and phospho-blocking T584V fusions with Ppc89.3Pk (detected with the 336 monoclonal antibody that recognizes the Pk epitope). IP, immunoprecipitation. (E) Yeast two-hybrid assays using the indicated baits and prey suggest that phosphorylation switches the affinity of unphosphorylated Sid4 for Ppc89 to an affinity for C*K1δ^Hhp1^/CK1δ^Hhp2^*. *n* = 3. For full dilution series, see Fig. S1 C. SD-L-T, SD-Leu-Trp; SD-L-T-H, SD-Leu-Trp-His.

We next wanted to address the means by which NIMA^Fin1^ phosphorylation of T584 could alter Sid4 function. As the phosphorylation site sits within the carboxyl terminal domain, which binds to the core SPB component Ppc89 (Rosenberg et al., 2006), we asked whether SPB association of Sid4 was influenced by T584 phosphorylation. We exploited an assay developed for the budding yeast Sid4 homologue Cnm67 in which fusion to the C-terminal domain directs GFP to the SPB (Fig. 3 A; Klenchin et al., 2011). Induction of analogous fusion proteins in which GFP was fused to residues 466–660 of Sid4 gave an SPB signal (Fig. 3 B). The signal persisted when 584 was mutated to valine, but it was abolished by mutation to either glutamic or aspartic acid to mimic phosphorylation (Fig. 3 C). Precipitation of these fusion proteins with GFP-Trap (Rothbauer et al., 2008) confirmed that Ppc89 association with the wild-type Sid4 fragment was disrupted by phosphomimetic T584D and T584E (Fig. 3 D). This disruptive impact of the phosphomimetic mutations was reiterated in the established ability of the C-terminal fragment of *sid4* (466–660 aa) to interact with *ppc89*^+^ in a yeast two-hybrid assay (Figs. 3 E and S1 C; Rosenberg et al., 2006). Significantly, although mutation of T584 to valine had no impact upon the strength of the two-hybrid interaction, the association of T584A was compromised to suggest that valine more accurately represents the nonphosphorylated state of T584 than alanine. We conclude that T584 phosphorylation regulates Sid4 affinity for Ppc89.

**Figure 4.**
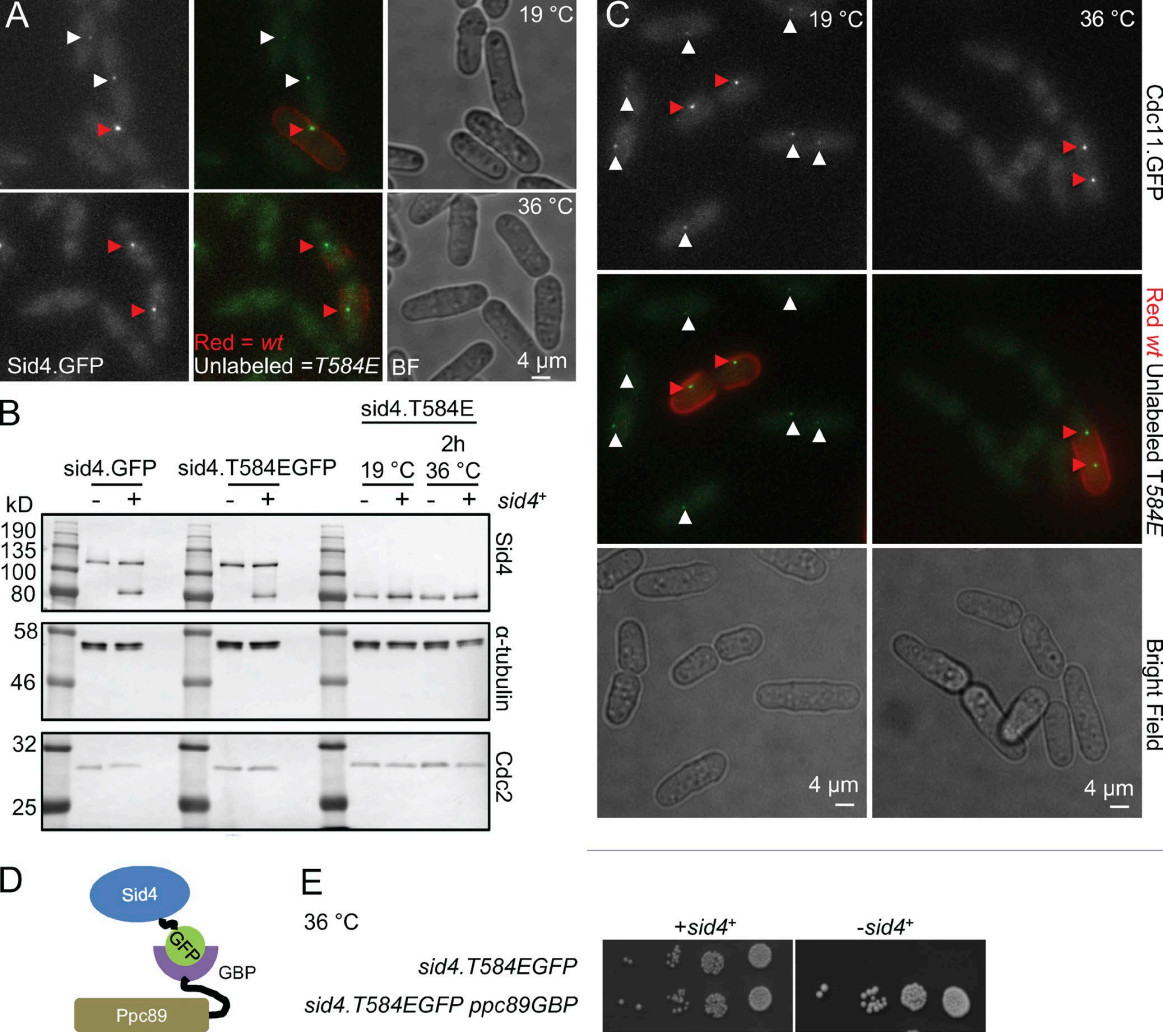
***sid4.T584E* compromises anchorage of Sid4 and Cdc11 to the SPB.** (A) Representative fluorescence images showing how the SPB affinity of the T584 phosphomimetic Sid4.T584EGFP fusion protein is reduced at 36°C. The cell walls of wild-type *sid4.GFP* cells were stained by resuspension in red lectin before being mixed with unstained *sid4.T584EGFP* cells and mounting the mixture for capture of a series of slices in the z axis that were merged to give the maximum projection shown. Red arrowheads indicate signals at SPBs of wild-type cells, and white arrows indicate signals at SPBs of *sid4.T584E* cells. *n* = 3. BF, brightfield. (B) Western blots of mid-log phase cultures of the indicated strains in which the transcription of an ectopic copy of *sid4*^+^ at the *hph.171* locus was either repressed (Sid4^−^) by the inclusion of 20 µM thiamine or de-repressed by the removal of thiamine 24 h before sampling (Sid4^+^). *n* = 3. (C) Representative fluorescence images of Cdc11.GFP in the indicated strains at the temperatures shown. Both fields show a mixture of *sid4*^+^ *cdc11.GFP* and *sid4.T584E cdc11.GFP* cells. The *sid4*^+^ *cdc11.GFP* cells were stained by transient resuspension in red fluorescent lectin to identify them in the mixed field of view to highlight the reduction in fluorescence intensity arising from the *sid4.T584E* mutation. *n* = 3. (D) The scheme for the anchorage of Sid4.T584EGFP to the SPB for the spot tests in E that show that *sid4.T584E* sin^−^ lethality arises from Sid4 departure from the SPB. (E) Spot tests in which serial dilution of the indicated strains were placed onto agar plates and incubated at 36**°**C.

Fusion of sequences encoding a GFP tag to the 3′ end of the *sid4.T584E* ORF generated a Sid4.T584EGFP fusion protein whose SPB association was notably diminished at 19°C and completely absent at 36°C (Fig. 4 A), even though *sid4.T584E* protein levels were unaffected by the shift from 19°C and 36°C (Fig. 4 B). As the amino terminus of Sid4 anchors the SIN to the SPB (Morrell et al., 2004), it seemed likely that the *sid4.T584E* sin^−^ phenotype arose from the inability to recruit the SIN to the SPB (Fig. 2, B and C). Certainly, the recruitment of the SIN component Cdc11.GFP mirrored the behavior of Sid4.T584EGFP in failing to bind the SPB at 36°C and associating weakly at 19°C (Fig. 4 C). We therefore asked whether we could restore SIN function to Sid4.T584E protein by artificially anchoring Sid4.T584E to the SPB by combining *sid4.T584EGFP* with a *ppc89.GBP* allele in which the sequences encoding the single-chain llama antibody GFP-binding protein (GBP; Rothbauer et al., 2006, 2008) were fused to the C terminus of Ppc89 (Fig. 4 D). Strikingly, these *sid4.T584EGFP ppc89.GBP* cells were viable at 36°C (Fig. 4 E). We conclude that *sid4.T584E* loss of viability does indeed arise from sin^−^ deficiency generated by the inability to recruit Sid4 to the SPB. This rescue experiment provides the first concrete noncorrelative evidence that anchorage to the SPB is essential for SIN function (Simanis, 2015).

### Recruitment of casein kinase 1δ (CK1δ) to Sid4, not departure of Sid4 from the SPB, suppresses *cut12.1*

**Figure 5.**
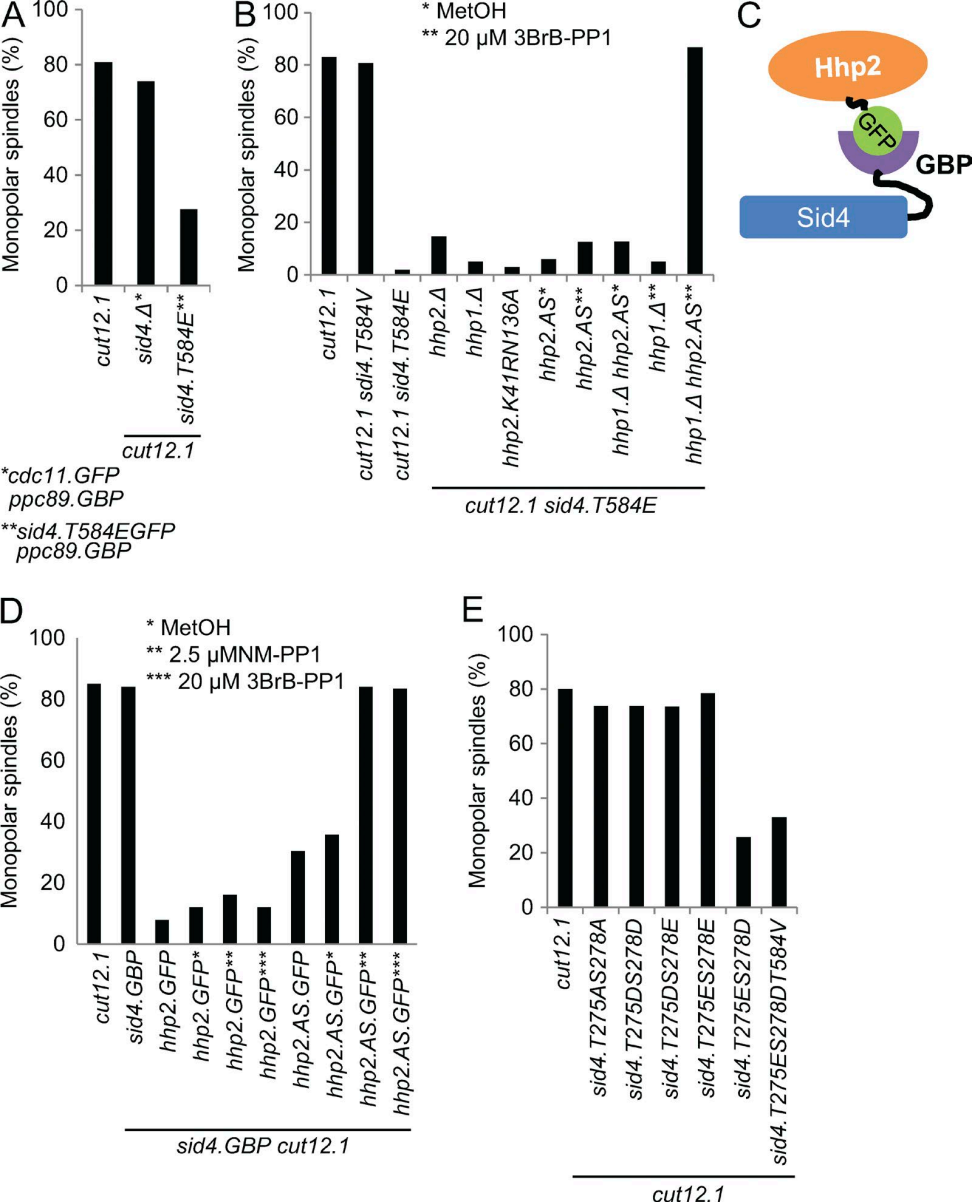
**Recruitment of CK1δ^Hhp1^/CK1δ^Hhp2^ to T584-phosphorylated Sid4 suppresses *cut12.1* monopolarity.** (A, B, D, and E) Representative monopolar spindle counts of the indicated strains as for Fig. 1 C. In each case, *n* = 3. See also Fig. S2. (C) The scheme for the anchorage of CK1δ^Hhp1^ and CK1δ^Hhp2^.AS to the SPB to suppress the spindle activation defect of *cut12.1*.

We next asked whether *cut12.1* suppression by *sid4.T584E* was also a consequence of Sid4 departure from the SPB. Combining *sid4.T584EGFP ppc89.GBP* with *cut12.1* showed that suppression was maintained when Sid4.T584EGFP was anchored to the SPB (Fig. 5 A). In a second approach, we asked whether complete removal of Sid4 from the cell would suppress *cut12.1*. To bypass the essential requirement for Sid4, we directly anchored the SIN scaffold Cdc11.GFP to SPB-associated Ppc89.GBP (Fig. S2 A) before deleting *sid4*^+^ from this background (*sid4.Δ*). Introduction of *cut12.1* to this *cdc11.GFP ppc89.GBP sid4.Δ* background confirmed that *cut12.1* suppression did not arise from the failure of Sid4.T584E to bind the SPB, and so the suppression of *cut12.1* must be the consequence of a distinct property conferred upon Sid4 by this phosphomimetic mutation (Fig. 5 A).

Johnson et al. (2013) proposed that events at the C terminus of Sid4 recruit CK1δ^Hhp1^ and CK1δ^Hhp2^. As mutation of T584 had a profound impact upon cell fate, we tested the association between the sequences encoding the C-terminal fragment of Sid4 (466–660 aa) and those encoding Hhp1 and Hhp2 in the yeast two-hybrid assay. A robust interaction between Sid4 and both *hhp1*^+^ and *hhp2*^+^ was detected when T584 was mutated to the phosphomimetic residues E/D (Figs 3 E and S1 C), whereas wild-type or T584V sequences failed to promote viability. Sid4.T584A exhibited a strong interaction with *hhp1*^+^ and a weak interaction with *hhp2*^+^ to consolidate the view that valine rather than alanine is an appropriate mutation to mimic the nonphosphorylated state of T584.

If the phosphomimetic Sid4.T584E protein suppresses *cut12.1* through the inappropriate recruitment of CK1δ^Hhp1^/CK1δ^Hhp2^, then removal of these casein kinases should abrogate this suppression. Given the robust functional redundancy between CK1δ^Hhp1^ and CK1δ^Hhp2^ (Ishiguro et al., 2010), it was not surprising that individual deletion of either *hhp1*^+^ or *hhp2*^+^ had no impact upon *cut12.1* suppression by *sid4.T584E* (Fig. 5 B). We therefore inhibited an analogue-sensitive Hhp2.AS kinase in an *hhp1.Δ* deletion background (Gregan et al., 2007). Strikingly, suppression of the monopolar spindle defect of *cut12.1* by *sid4.T584E* was fully reversed by ablation of CK1δ activity (Fig. 5 B).

To confirm that it is indeed the recruitment of CK1δ by Sid4.T584E that confers *cut12.1* suppression, we recruited CK1δ^Hhp2^.GFP to an otherwise wild-type Sid4.GBP anchor on the SPB. Not only did this surrogate recruitment of CK1δ^Hhp2^.GFP to Sid4.GBP confer robust suppression of *cut12.1* (Figs. 5 D and S2 B), but it was dependent on kinase activity because suppression was analogue sensitive when the version of CK1δ^Hhp2^ that was recruited incorporated the analogue-sensitizing mutation (Figs. 5 D and S2 B). We conclude that *sid4.T584E* suppression of *cut12.1* arises from the inappropriate recruitment of CK1δ activity to Sid4.

### CK1δ-directed phosphorylation of Sid4 on both T275 and S278 suppresses *cut12.1*

Johnson et al. (2013) showed how Sid4 phosphorylation at positions T275 and S278 by CK1δ^Hhp1^/CK1δ^Hhp2^ recruits the ubiquitin ligase Dma1 to block septation when spindle function is perturbed. To determine whether T275 and S278 phosphorylation was the means by which CK1δ^Hhp1^/CK1δ^Hhp2^ recruitment to Sid4 suppressed *cut12.1*, we tested all permutations of phosphomimetic mutations of the endogenous locus T275 and S278 for *cut12.1* suppression. *Sid4.T275ES278D* suppressed the monopolar spindle defect and lethality of *cut12.1* (Figs. 5 E and S2, C and D). Importantly, these phosphomimetic mutations bypassed the requirement for T584 phosphorylation as *sid4.T275ES278DT584V* at the endogenous locus suppressed *cut12.1* (Fig. 5 E).

**Figure 6.**
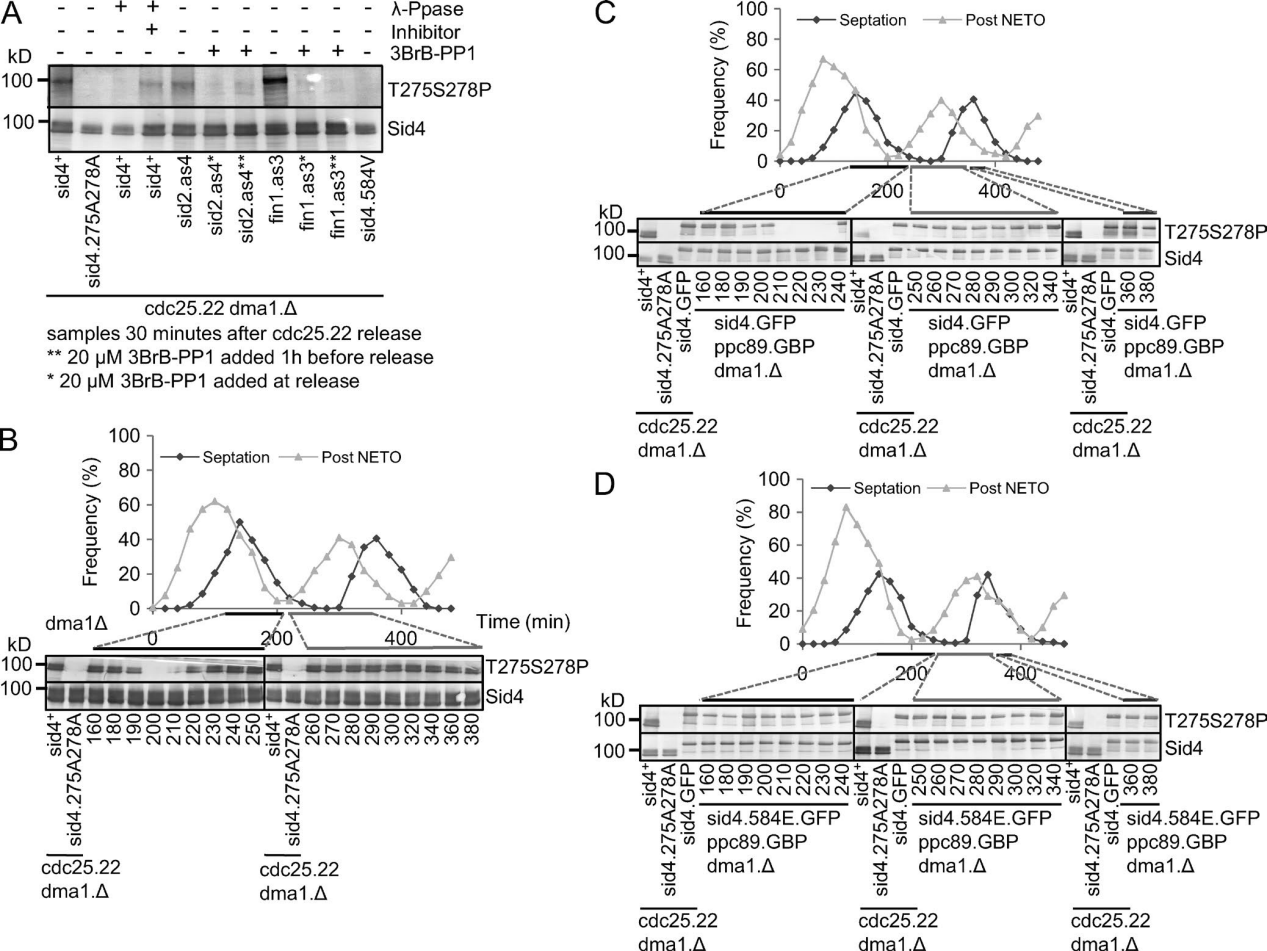
**Phosphorylation on T584 promotes cell cycle–dependent phosphorylation of both T275 and S278.** (A) Antibodies raised against a peptide corresponding with Sid4 simultaneously phosphorylated on T275 and S278 used to blot Sid4 preparations immunoprecipitated from denatured extracts of the indicated strains. Immunoprecipitates were incubated for 30 min at 30°C with λ phosphatase, its inhibitor, or buffer alone, as indicated. (B–D) Small G2 *dma1.Δ* (B), *sid4.GFP ppc89.GBP dma1.Δ* (C), or *sid4.T584EGFP ppc89.GBP dma1.Δ* (D) cells were isolated from mid-log phase cultures by centrifugal elutriation at *t* = 0, and aliquots were taken at the indicated intervals to monitor septation and new end take off (NETO) with calcofluor white or phosphorylation on both T275 and S278. Control samples 40 min after *cdc25.22 dma1.Δ* control cultures were released from 4.25 h arrest at 36**°**C as indicated.

Antibodies that only recognize Sid4 when phosphorylated on both T275 and S278 (Fig. 6 A) revealed a T275S278 phosphorylation signal that began in G2 to persist until the peak of septation (Fig. 6 B). T275S278 dual phosphorylation was dependent on T584 phosphorylation because it was absent from *sid4.T584V* cells (Fig. 6 A) and yet was constitutively present throughout *sid4.T584EGFP ppc89.GBP* cell cycles (Fig. 6, C and D). We conclude that *sid4.T584E* promotes inappropriate phosphorylation of Sid4 on both T275 and S278 and that it is this phosphorylation that suppresses the mitotic commitment defect of *cut12.1*.

### Recruitment of Chk2^Cds1^ to T275S278-phosphorylated Sid4 suppresses *cut12.1*

**Figure 7.**
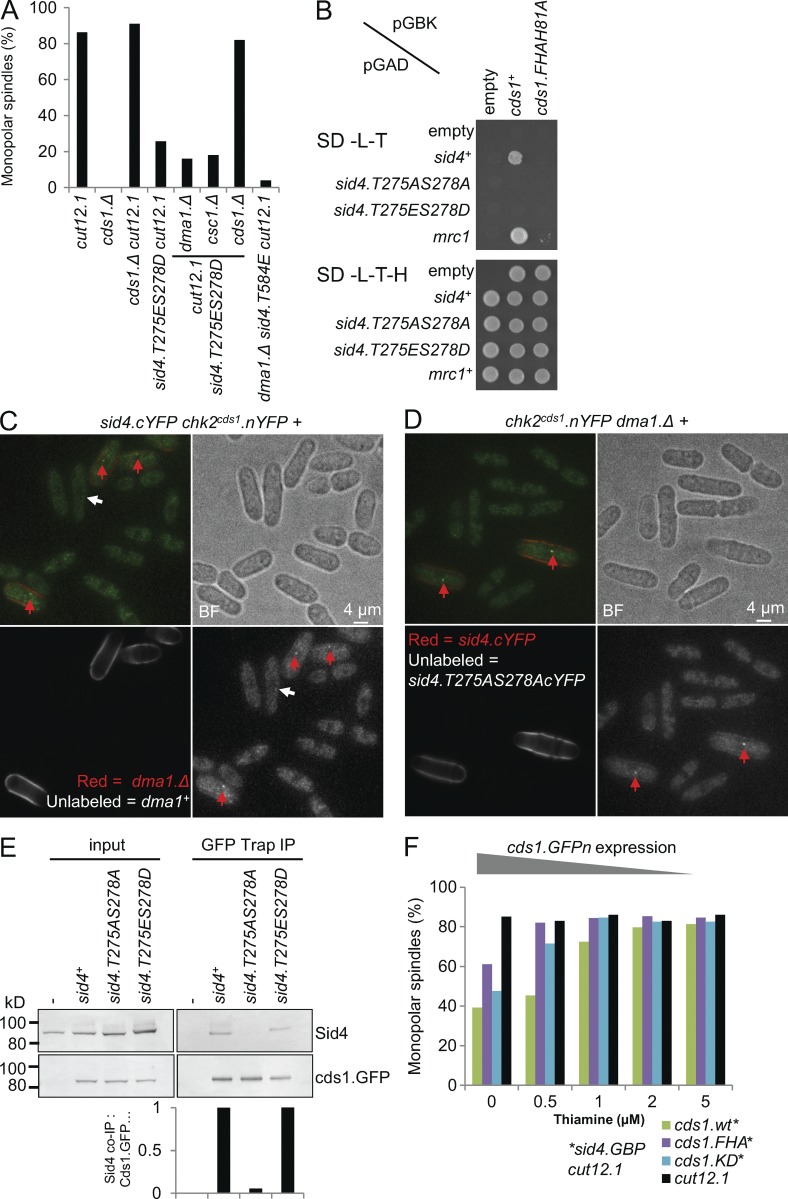
**Recruitment of Chk2^Cds1^ to T275S278-phosphorylated Sid4 compensates for the SPB activation defect of c*ut12.1*.** (A and F) Representative plots of monopolar spindle counts as for Fig. 1 C. See also Fig. S3 A. In each case, *n* = 3. (B) Yeast two-hybrid comparisons of the indicated constructs. For full dilution series, see Fig. S3 B. Mrc1 is a validated Chk2^Cds1^ partner (Tanaka and Russell, 2001). SD-L-T, SD-Leu-Trp; SD-L-T-H, SD-Leu-Trp-His. (C and D) BiFc assays of fluorescence generated between Chk2^Cds1^.nYFP and Sid4.cYFP in the indicated strains. Each field shows cells from cultures of different strains as indicated. Cell walls of the strain named in red having been stained by transient suspension in red fluorescent lectin to identify it in the mixed field. The images are maximum projections of deconvoluted z stacks that span the diameter of the cell. See also Fig. S3 C. *n* = 3. Red arrows indicate signals at SPBs of wild-type *sid4^+^* cells, and white arrows indicate signals at SPBs of *sid4.T275A278AcYFP* cells. BF, brightfield. (E) GFP-Trap precipitates from the indicated Chk2^Cds1^.GFP d*ma1.Δ* strains probed with Sid4 antibodies to detect coprecipitating Sid4. Coprecipitation was abolished by simultaneous phospho-blocking mutation at 275 and 278. *n* = 3. IP, immunoprecipitation. (F) A switch from 20 µM thiamine to the indicated concentrations 24 h before the temperature shift to 36**°**C de-repressed transcription of the Chk2^Cds1^.GFPn in a dose-dependent manner. *n* = 3.

Because T275S278 phosphorylation generates a docking site for the forkhead-associated (FHA) domain of Dma1, we asked whether *sid4.T584E* and *sid4.T275ES278D* suppressed *cut12.1* because they inappropriately recruited Dma1 to Sid4 (Johnson et al., 2013). Unexpectedly, *dma1*^+^ deletion had no impact upon *cut12.1* suppression (Figs. 7 A and S3 A). Testing the other FHA domain proteins Csc1 (Singh et al., 2011) and Chk2^Cds1^ (Murakami and Okayama, 1995; Boddy et al., 1998; Lindsay et al., 1998) for an impact upon *cut12.1* suppression revealed a reliance upon *chk2^cds1^* (Figs. 7 A and S3 A). A positive reaction between the FHA domain of Chk2^Cds1^ and Sid4 in the yeast two-hybrid assay was abolished by mutation of either *chk2^cds1^* FHA domain (H81A; Tanaka and Russell, 2001) or *sid4.T275AS278A* (Figs. 7 B and S3 B). We therefore used the bimolecular fluorescence complementation (BiFc) technique to monitor the association between Sid4 and Chk2^Cds1^ in vivo. In this assay, two halves of YFP are fused to candidate proteins. If these partners reside within a common complex, the two halves can dock to generate a functional fluorescent protein (Hu et al., 2002). BiFc analysis with Chk2^Cds1^:nYFP and Sid4.cYFP gave a dim SPB signal whose intensity increased markedly when *dma1.Δ* removed competition for T275S278 docking from Dma1 (Fig. 7 C). Importantly, no signal was detected when T275S278 could not be phosphorylated to create an FHA anchor in *sid4.T275AS278AcYFP chk2^cds1^.nYFP dma1.Δ* cells (Figs. 7 D and S3 C). Coimmunoprecipitation confirmed the association between Chk2^Cds1^ and Sid4 as Chk2^Cds1^.GFP precipitated with wild-type Sid4 and Sid4.T275ES278D but not with Sid4.T275AS278A (Fig. 7 E).

We next asked whether it was the recruitment of Chk2^Cds1^ to phosphorylated Sid4 that suppressed the mitotic SPB activation defect of *cut12.1*. As permanent anchorage of Chk2^Cds1^.GFP to Sid4.GBP compromised fitness (not depicted), we used the thiamine-repressible *nmt81* promoter to induce different levels of *chk2^cds1^.GFP* expression in a *sid4.GBP cut12.1* background. The failure to activate the new SPB was suppressed in a dose-dependent manner (Fig. 7 F).

### Chk2^Cds1^-directed eviction of Flp1 from the SPB suppresses cut12.1

**Figure 8.**
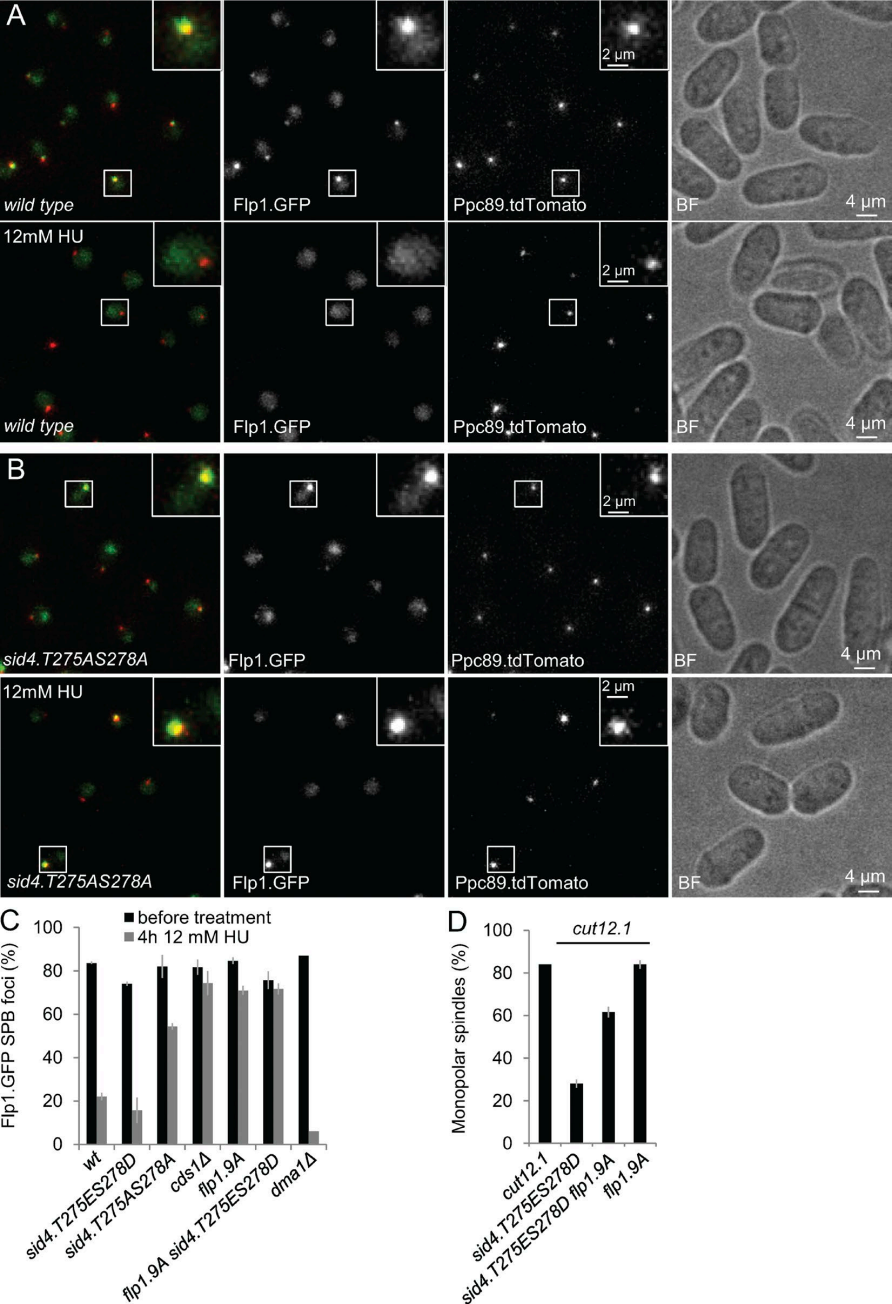
**Blocking phosphorylation on T275 and S278 of Sid4 to block Chk2^Cds1^ recruitment abolishes Chk2^Cds1^’s ability of to evict Flp1.GFP from the SPB.** (A and B) Representative fluorescence and brightfield (BF) images of *flp1.GFP ppc89.tdTom* cells that reveal how the eviction of Flp1.GFP from the SPB is abolished in the *sid4.T275AS278A* background that prevents Chk2^Cds1^ recruitment to the SPB. (C) Quantification of the data in A and B. *n* = 3. For controls, see Fig. S4. (D) Frequency of monopolarity in the indicated strains as for Fig. 1 B. Error bars in C and D indicate SD for three independent experiments.

Hydroxyurea (HU) inhibits ribonucleotide reductase to deplete the nucleotides required for DNA replication. HU treatment prompts Chk2^Cds1^ to evict the Cdc14 family phosphatase Flp1/Clp1 from the SPB (Díaz-Cuervo and Bueno, 2008; Broadus and Gould, 2012). Cdc14 family phosphatases dephosphorylate the serine proline/threonine proline motifs that are targeted by Cdk1–cyclin B (Mocciaro and Schiebel, 2010). Furthermore, *Schizosaccharomyces pombe* Flp1/Clp1 inactivates Cdc25 (Esteban et al., 2004; Wolfe and Gould, 2004). Thus, Chk2^Cds1^ eviction of Flp1 from the pole is an attractive mode for suppression of *cut12.1* by Sid4 because it would act at two levels to reduce the local threshold for Cdk1–cyclin B activation at the SPB. We therefore monitored the distribution of Flp1.GFP in strains that bore simultaneous mutation of T275 and S278 after HU addition to determine whether the kinase relay on Sid4 we have defined underpins Flp1 eviction. Strikingly, the phospho-blocking *sid4.T275AS278A* mutation emulated the impact of Chk2^Cds1^ removal in abolishing the HU-invoked eviction of Flp1.GFP from the spindle poles (Figs. 8 and S4). Importantly, the *flp1.9A* allele that blocks Chk2^Cds1^-mediated eviction from the SPB (Díaz-Cuervo and Bueno, 2008) compromised the ability of *sid4.T275ES278D* to suppress the Cdk1–cyclin B activation defect of *cut12.1* (Figs. 8 D and S4). We conclude that the phosphorylation of Sid4 at position T584 recruits CK1δ^Hhp1^/CK1δ^Hhp2^ to promote an association of Chk2^Cds1^ with Sid4 that ejects Flp1 from the SPB.

## Discussion

**Figure 9.**
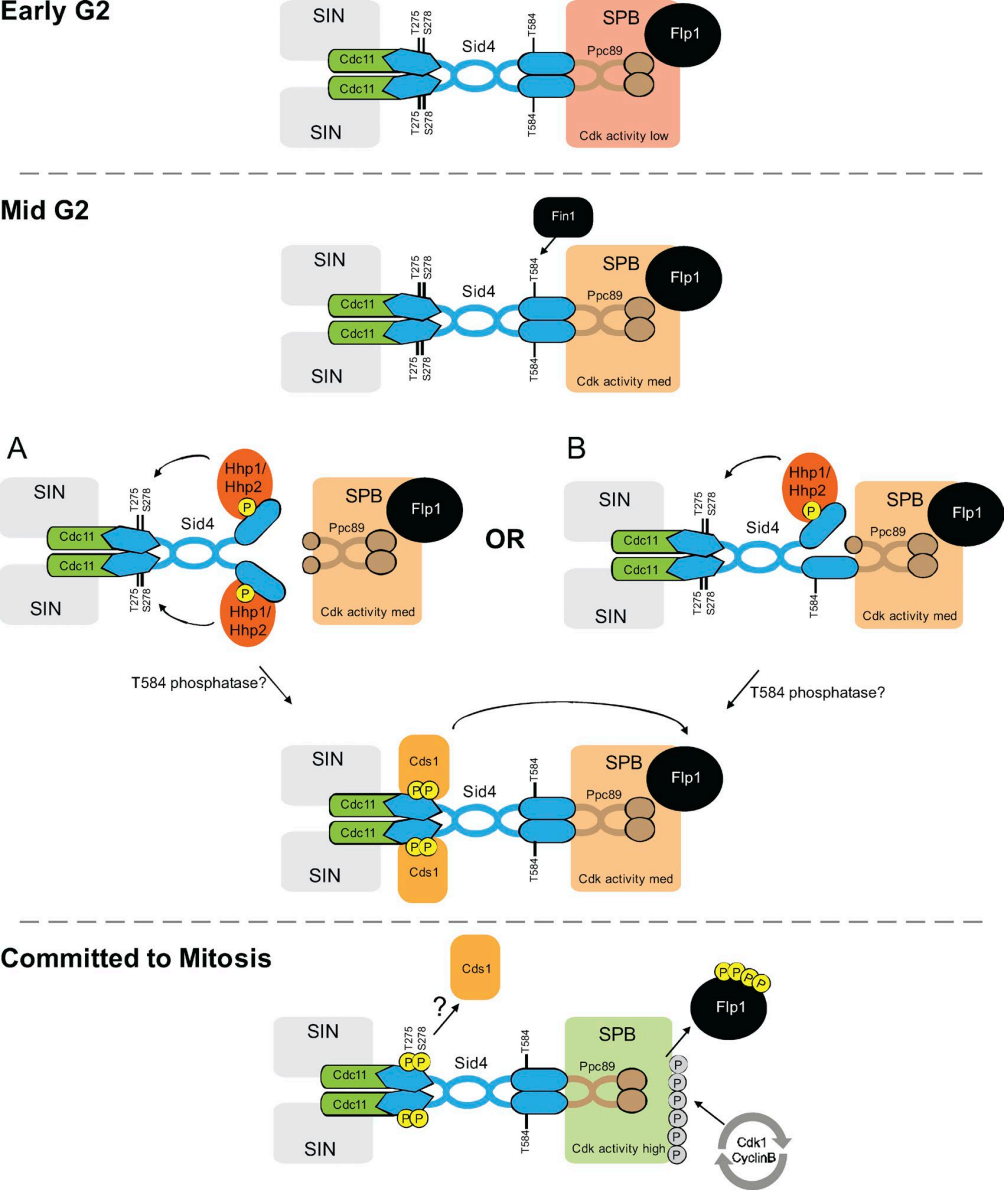
**Model: Flp1 eviction by a Sid4-mediated NIMA^Fin1^, CK1δ^Hhp1^/CK1δ^Hhp2^, Chk2^Cds1^ relay to boost the impact of Cdk1–cyclin B activation at the SPB.** Phosphorylation of T584 by NIMA^Fin1^ reduces Sid4 affinity for Ppc89 and supports binding to CK1δ^Hhp1^/CK1δ^Hhp2^. The CK1δ kinases then phosphorylate T275 and S278 to promote the recruitment of Chk2^Cds1^. As we see a BiFc signal between Chk2^Cds1^ and Sid4 that is sensitive to competition from Dma1 on the SPB (Fig. 7, C and D; and Fig. S3 C), we assume that the loss of affinity for Ppc89 on the SPB (A) is rapidly followed by a dephosphorylation event after CK1δ kinases have phosphorylated T275 and S278 to create the docking site for Chk2^Cds1^. Alternatively, Sid4 anchorage to the SPB is retained while CK1δ kinases phosphorylate T275 and S278. This could be acheived by the phosphorylation of only one Sid4 molecule within a Sid4 dimer (B) or through more complex higher-order associations of Sid4 molecules that await characterization. The T584 phosphatase that would be an essential component in A could equally well operate in the scheme shown in B to remove phosphate from T584 while Chk2^Cds1^ is anchored to Sid4 phosphorylated on T275 and S278. Sid4-anchored Chk2^Cds1^ phosphorylates Flp1 phosphatase to reduce its affinity for the SPB, thereby lowering antagonism toward Cdk1–cyclin B phosphorylation events on the SPB. The consequence of this cascade is a reduction in the threshold of phosphorylation that must be passed in order to convert the SPB into a mitotic state. The SPB matures from an early G2 state with no potential to invoke mitosis (top; red SPB) to a state with all three kinases in cycles of activation on the SPB (middle; amber) to the mitotic commitment state of Flp1 expulsion from the SPB (bottom; green). We assume that the tipping of the balance between mid-G2 and commitment arises from alterations in the phosphatase activities that dephosphorylate T584, T275, and S278.

We show how a phosphorylation cascade along Sid4 is able to compensate for Cdk1–cyclin B activation defects arising from the *cut12.1* mutation. The chain of events is initiated by NIMA^Fin1^ phosphorylation of T584 to promote CK1δ recruitment (Fig. 9). CK1δ phosphorylation generates a docking site for the FHA domain of Chk2^Cds1^, from which Chk2^Cds1^ is able to execute its characterized role of evicting the Cdc14 phosphatase from the SPB (Díaz-Cuervo and Bueno, 2008; Broadus and Gould, 2012). Dephosphorylation of Cdc25 by Flp1 reduces Cdc25 activity (Esteban et al., 2004; Wolfe and Gould, 2004), and Cdc14 family phosphatases dephosphorylate the serine proline/threonine proline motifs that are targeted by Cdk1–cyclin B (Mocciaro and Schiebel, 2010). Thus, Flp1 eviction will reduce the local threshold for Cdk1–cyclin B on the new SPB to support activation of the defective new SPB of *cut12.1* cells (Figs. 1 B and 9).

This dialog between two independent scaffolds reinforces the robustness of the mitotic switch. Activation of NIMA^Fin1^ impacts at two points in this core switch: the eviction of PP1 from Cut12 (Grallert et al., 2013b) and the recruitment of Chk2^Cds1^ to Sid4. NIMA^Fin1^ also promotes mitotic commitment via a third, less-defined route through the Pom1 cell geometry network (Grallert et al., 2012). As protein kinases, CK1δ^Hhp1^, CK1δ^Hhp2^, and Chk2^Cds1^ each have the potential to emulate NIMA^Fin1^ in hitting further targets in the switch to enhance the robustness and bistable nature of the mitotic commitment switch.

The concept of dialog between scaffolding proteins on the SPB addresses the longstanding question as to why mitotic entry and exit should be regulated from the spindle pole. Centrosomal scaffold dialog can integrate inputs from diverse signaling networks with a limited number of neighboring molecules in order to generate a single coherent output that can then be amplified throughout the cell. Alongside centrosomal signaling in cell cycle and DNA checkpoint controls, the overriding impact of the spindle assembly checkpoint signal emanating from a single kinetochore provides another example of the dramatic impact that coordinated singling from a single center can have (Lara-Gonzalez et al., 2012).

Sid4 is a classic example of a centrosomal scaffold that sits at the intersection of multiple signaling pathways. Sid4’s role in recruiting Cdc11 to anchor the SIN is well established (Krapp et al., 2001; Tomlin et al., 2002). The activation of Sid4-anchored SIN signaling on the newer of the two SPBs late in anaphase B triggers cytokinesis and mitotic exit (Sohrmann et al., 1998; Grallert et al., 2004). In addition to driving the recruitment of Chk2^Cds1^ in the pathway that sets the level of Cdk1–cyclin B activity at the SPB, which we define in this study, dual phosphorylation at T275 and S278 independently recruits Dma1 in the distinct control that maintains mitotic arrest in response to spindle microtubule perturbation (Tomlin et al., 2002; Johnson and Gould, 2011; Johnson et al., 2013). Our demonstration that Sid4 anchors Chk2^Cds1^ to expel Flp1 from the SPB when DNA replication is incomplete now puts Sid4 into DNA checkpoint signaling in an echo of the role played by pericentrin, CDK5RAP2, and AKAP450 in checkpoint signaling in human cells (Griffith et al., 2008; Barr et al., 2010).

We believe that further studies will reveal similar networks in mitotic controls in other systems as both CK1δ and Chk2 are recruited to human centrosomes (Sillibourne et al., 2002; Tsvetkov et al., 2003). Although it is clear that CK1δ activity regulates the timing of mitotic commitment of human cells by targeting Wee1 for destruction (Penas et al., 2014) before itself being degraded by the APC/C^Cdh1^ (Penas et al., 2015), it remains to be established whether it is this centrosomal pool of CK1δ that mediates these controls. Chk2 is activated in mitosis independently of any stimulation from its role in DNA damage responses (Stolz et al., 2010). As for fission yeast Chk2^Cds1^, Chk2 association with *Drosophila melanogaster* centrosomes is reliant upon the function of its FHA domain (Takada et al., 2015). Critically, phosphorylation of human BRCA1 by Chk2 recruits PP6-SAP3 to counteract the activation of Aurora A at centrosomes (Stolz et al., 2010; Ertych et al., 2014, 2016). The abnormal enhancement of centrosomal Aurora A that arises from Chk2 depletion elevates microtubule dynamics to promote chromosome loss in the classic chromosome instability phenotype associated with tumors (Ertych et al., 2014, 2016). Strikingly, Aurora kinase also plays a key role in triggering the Cut12-mediated activation of polo at the fission yeast SPB at the core of the mitotic commitment controls addressed in our study (Petersen and Hagan, 2005; Petersen and Nurse, 2007; Hálová and Petersen, 2011). 

Such extensive similarities between fission yeast and human centrosomal CK1δ/Chk2/Aurora A/Polo signaling define some simple dependency relationships that can be probed in higher systems to unravel the complex usage of the spindle pole as a hub at which to coordinate signaling events that determine cell fate.

## Materials and methods

### Yeast culture and growth

Strains used are listed in Table S1. *cdc11.GFP*, *sid4.gfp*, *flp1.gfp*, and *sid4.myc13* were gifts from V. Simanis (École Polytechnique Fédérale de Lausanne, Lausanne, Switzerland), *hhp2.GFP-UTR:kan^R^* was from J. Gregan (Comenius University, Bratislava, Slovakia), and *cds1::ura4^+^* was from A.M. Carr (University of Sussex, Brighton, England, UK). Cell culture and maintenance (Petersen and Russell, 2016) was performed as follows. Unless specified, yeast strains were streaked on yeast extract supplemented with adenine, histidine, leucine, uracil, and lysine (YES) plates from frozen glycerol stocks at −80°C and grown at a permissive temperature (25°C) until single colonies were formed. One single colony was then used to start overnight starter liquid cultures in Edinburgh minimal media 2 (EMM2) and grown at 25°C. Main cultures were inoculated from overnight starter liquid cultures and allowed to grow for at least 17 h (approximately three generations) at 25°C. *sid4.T584E* mutants were streaked on YES plates and grown at 20°C, and for starter and liquid culture experiments, cells were grown in EMM2 with 40 µg/ml thiamine at 19°C.

Centrifugal elutriation was used to isolate small G2 cells (Hagan et al., 2016). In brief, a 1-liter starter culture of each prototrophic strain was diluted to generate 7 liters of culture at a density of 3.8 × 10^6^ 19 h later. These 7 liters were loaded into the 40-ml chamber of a Beckman Elutriator JE-5.0 rotor in a modified J6 centrifuge over 27 min. 200 ml of media was collected from the exhaust tube 23 min after loading began. Incremental reduction of the centrifuge speed eluted small G2 cells from the asynchronous population in the chamber. During elutriation, the 200 ml of exhaust media was added to the media flowing through the chamber. The freshly eluted G2 cells were immediately diluted into prewarmed EMM2 to generate a 2.5-liter culture at a cell density of 10^6^ cells/ml. The synchrony of cell division in the culture was monitored by scoring the septation index through staining with the fluorescent cell wall stain calcofluor white (Hagan, 2016a).

For protein induction using the thiamine-repressible *nmt1* derived promoters (Maundrell, 1990), cells were grown at early log phase (10^6^ – 2 × 10^6^ cells/ml) at 25°C in EMM2 with 20 µM thiamine before being harvested via centrifugation at 1,300 *g* for 2 min. The cell pellets were subsequently washed with 10 ml of EMM2 without thiamine. After two further washes, cells were inoculated into EMM2 without thiamine. Cells were subsequently grown at 25°C for 24 h before analysis.

For the experiments with attenuated *cds1.GFPn* expression levels, 5 µM, 2 µM, 1 µM, 0.5 µM, or no thiamine was added back to the culture after the thiamine washes, and cultures were grown for a further 24 h at 25°C before being shifted to 36°C.

For the inhibition of analogue-sensitive kinases, ATP analogues 1NM-PP1 and 3BrB-PP1 (Toronto Research Chemicals; Dalton Pharma Services) were dissolved in methanol to generate 10-mM and 50-mM stock solutions, respectively, before addition to media at the concentrations specified in the figure legends.

### Live-cell imaging

All strains used for live-cell imaging were prototrophs. The starter and main cultures for live imaging of cells were both grown in filter-sterilized EMM2. Unless specified, the cultures were grown at 25°C to a cell density of 10^6^ cells/ml before being harvested via centrifugation at 1,800 *g* for 2 min at room temperature and resuspended in 500 µl of EMM2. 10 µl of cells were mixed with plant lectin (5 mg; L1395; Sigma-Aldrich) to a final concentration of 0.1 mg/ml and attached onto glass coverslips for 3 min before the coverslips were washed three times with 1 ml of EMM2 to remove any unattached cells. The coverslips were subsequently mounted in an FCS2 chamber (Bioptechs). All live imaging was conducted on a DeltaVision Core system (Applied Precision Ltd.) fitted with a 100× 1.45 NA objective (ZEISS), a Cascade II:1024 electron-multiplying charge-coupled device camera (Photometrics), and a 89000 ET-Sedat Quad filter set (Chroma Technology Corp.) and controlled by SoftWoRx software (Applied Precision Ltd.). Images shown are maximal projections of sections composed of 30 slices (0.3 µm apart) that were taken and processed with Imaris software (Bitplane). For the BiFc images that monitored the interaction between Cds1.nYFP and Sid4.cYFP, individual slices of image stacks were digitally deconvolved using Huygens Remote Manager (Ponti et al., 2007), and maximal projections of the deconvolved image stacks were generated with Imaris software.

For TRITC-lectin labeling of live cells, 100 µl of cells was labeled with 0.01 mg/ml TRITC-conjugated lectin (2 mg; L5264; Sigma-Aldrich) in filter-sterilized EMM2 at room temperature for 5 min. The cells were subsequently centrifuged at 850 *g* for 2 min and washed three times with filter-sterilized EMM2. Labeled and unlabeled cells were mixed with a ratio of 1:1, and 10 µl of this cell mixture was attached onto glass coverslips as described in the prior paragraph.

### Immunofluorescence

Tubulin and Sad1 staining were conducted using established procedures (Hagan and Hyams, 1988; Hagan, 2016b). Cells were fixed by the addition of freshly prepared prewarmed 30% formaldehyde solution in PEM (100 mM piperazine-*N*,*N*-bis(2-ethanesulfonic acid) [Pipes and sodium salt], 1 mM EGTA, and 1 mM MgSO_4_, pH 6.9) to the culture to a final concentration of 3.7%. 30 s later, glutaraldehyde was added to a final concentration of 0.2%. After fixation for 30–120 min and three washes in PEM, cells were washed once in PEMS (PEM and 1.2 M sorbitol) before digestion of the cell wall with zymolyase 100-T in PEMS. After permeabilization in PEM and 1% triton X-100 and then a further three washes in PEM, cells were resuspended in PEM and 1 mg/ml NaBH_3_ for 5 min, after which a further three washes in PEM were followed by resuspension in PEMBAL (PEM + 1% BSA [globulin free], 1% NaN_3_, and 100 mM lysine HCl) for 30 min. Cells were pelleted once more before resuspension in PEMBAL with affinity-purified polyclonal Sad1 antibodies (Hagan and Yanagida, 1995) at a concentration of 1:100 alongside tissue culture supernatant containing the TAT1 monoclonal antibody (a gift from K. Gull, University of Oxford, Oxford, England, UK; Woods et al., 1989) at a dilution of 1:80. Incubation overnight at room temperature with agitation was followed by three washes in PEMBAL and resuspension in PEMBAL containing appropriate secondary antibodies (FITC-conjugated goat anti–mouse specific IgG antibodies to detect TAT1 and Cy3-conjugated goat anti–rabbit specific IgG antibodies to recognize Sad1). After a further 5 h at room temperature with agitation, a final three PEMBAL washes were followed by a wash in PBS 1% NaN_3_ and resuspension in PBS 1% NaN_3_ and 0.2 µg/ml DAPI. Imaging was conducted on the DeltaVision core system described in the previous section. The representative images shown in the manuscript are maximum projections of sections composed of 30 slices (0.3 µm apart) that were taken and processed with Imaris software.

### Genetic manipulation

Mutant *sid4* alleles were generated via mutagenesis using the Phusion system (New England Biolabs, Inc.) and inserted into the genome using the *kanMX6* at the 3′ UTR as a marker immediately after the stop codon. For the split BiFc assay, *sid4^+^* was tagged with the C-terminal fragment of Venus YFP, whereas *cds1^+^* was tagged with the N-terminal fragment of Venus YFP (Grallert et al., 2013b). Both *sid4^+^* and *cds1^+^* loci were tagged at the C terminus using PCR tagging (Bähler et al., 1998; Grallert et al., 2013b). Sequences for the generation of GBP fusion proteins were obtained from G. Pereira (Ruprecht-Karls-Universität Heidelberg, Heidelberg, Germany) and H. Leonhardt (Ludwig-Maximilians-Universität München, Munich, Germany).

For the generation of *sid4.T584E* and *sid4.T275ES278D* phospho mutants, the TADH natMX6 or TADHkanMX6 markers that incorporate the terminator sequences of the budding yeast ADH gene before each marker, from the pFA6 series of tagging vectors were inserted at the STOP codon of mutated genomic *sid4* sequences. A fragment containing each mutant *sid4* ORF, the TADH, and marker within 400 bp of flanking sequences were transformed into a strain containing an additional copy of *sid4^+^* integrated into the *hph.171k* locus under the control of the thiamine-repressible promoter *nmt81* (Fennessy et al., 2014). All strains bearing *sid4* phospho mutant alleles were backcrossed at least three times, and prototrophs were isolated before use in experiments.

For the generation of stably integrated inducible ectopic genes, the gene of interest was cloned into the pINTH81 vector series via Nde1–BamH1 restriction sites and integrated into the *hph.171k* locus (Fennessy et al., 2014).

Generation of unmarked *flp1.9A* and *hhp2.as* alleles with native UTRs: The mutation conferring analogue sensitivity upon Hhp2 (M85A) was introduced via site-directed mutagenesis into sequences encoding the Hhp2 ORF amplified from genomic DNA alongside 250 bp of flanking DNA and Not1 sites. The *flp1.9A* construct was made by generating a synthetic gene (Thermo Fisher Scientific) in which all Chk2^Cds1^ consensus phosphorylation sites reported by Díaz-Cuervo and Bueno (2008) in the Flp1 sequence were mutated to alanine within 205 bp of flanking genomic regions straddled by Not1 sites (Díaz-Cuervo and Bueno, 2008). Each mutant gene, including the flanking 250-bp UTR, was excised on a Not1 fragment and transformed into an *rpl42.SP56Q S. pombe* host in which the ORF had been disrupted with *rpl42^+^*, and the integration event was selected for by plating on plates containing 100 µg/ml cycloheximide (Fennessy et al., 2014). The loci of cycloheximide-resistant mutants were amplified via PCR and sequenced for confirmation of gene sequences. Each mutant allele was backcrossed three times, and a prototroph without the *rpl42.SP56Q* mutation was isolated for use in experiments. The *flp1.9AGFP* allele was generated via PCR, tagging the *flp1.9A* allele with GFP at the C terminus (Bähler et al., 1998).

### Bacterial expression of full-length Sid4 and purification

The *sid4*^+^ ORF was cloned into pET41 vector and transformed into BL21 (C2527I; New England Biolabs, Inc.) competent *Escherichia coli* cells together with the pLysS vector (Studier et al., 1990). *E. coli* cells resistant to chloramphenicol and ampicillin were selected and grown in Luria-Bertani liquid medium at 37°C. When the OD at 600 nm reached 0.4, 1 mM IPTG was added to induce *sid4^+^* expression. Cells were harvested after 1 h of induction at 37°C. Sid4 was purified from inclusion bodies by separating the proteins of inclusion bodies in a 5-mm-thick 10% acrylamide SDS PAGE gel and excising the protein band corresponding with Sid4 before electroelution into elution buffer (16.8 mM Na_2_HPO_4_.12H_2_O, 11.4 mM NaH_2_PO_4_.2H_2_O, and 0.0288% SDS, pH 7.4).

### Antibody generation

Sheep polyclonal antibodies against Sid4 and GFP were raised by Diagnostics Scotland using full-length recombinant proteins purified from *E. coli* as the antigen. Affinity purification of sheep polyclonal antibodies were performed on beaded agarose gel columns containing covalently linked purified full-length Sid4^+^ or GFP peptide. The columns were generated using the AminoLink Plus Immobilization kit (44894; Thermo Fisher Scientific), and purification was performed according to the manufacturer’s instructions with the following modifications: purified antibodies were eluted in 900-µl fractions in elution buffer (0.5 M NaCl, 0.05 M glycine, and 100 µg/ml BSA, pH 2.3), and 100 µl of neutralization buffer (0.5 M Na_2_HPO_4_) was subsequently added to each fraction. A total of 10 fractions were collected. Fractions containing purified antibodies were identified via loading of 10-µl samples from each fraction on a 10% acrylamide SDS-PAGE gel and were then stained with Coomassie Blue. Fractions containing high concentrations of antibodies were pooled, and glycerol was added to a final concentration of 50% before being snap frozen using liquid N_2_ and then were stored at –80°C.

Rabbit polyclonal antibodies that specifically recognized peptides corresponding with Sid4 peptides when phosphorylated on T584 or simultaneously on both T275 and S278 were generated and affinity purified by Eurogentec.

### Yeast two-hybrid

Vectors, strains, and media used in the yeast two-hybrid assay were obtained from TaKaRa (Matchmaker Gold Yeast Two-Hybrid System; 630489). Full-length or fragments of *sid4^+^*, *cds1^+^*, *hhp1^+^*, *hhp2^+^*, and *ppc89^+^* were cloned into either into pGADT7 activation domain (AD) vector or pGBKT7 DNA-BD Vector via the Nde1 and BamH1 restriction sites. The pGADT7 AD vector-based constructs were transformed into Y187 yeast strain according to manufacturer’s instructions and selected in SD-Leu agar plates. The pGBKT7 AD Vector–based constructs were transformed into Y2HGold yeast strain according to the manufacturer’s instructions and selected in SD-Trp agar plates. Single colonies were isolated from each transformation, and crosses were made between Y187 and Y2HGold on YPD agar plates and grown overnight at 30°C to generate diploids. Diploids were selected using SD-Trp-Leu agar plates. To perform yeast two-hybrid, single colonies of diploid strains containing both pGBKT7 and pGADT7 constructs were grown for at least 4 h at 30°C in SD-Trp-Leu media before OD_600_ was used to calculate the plating of indicated cell numbers indicated on SD-Trp-Leu and SD-Trp-Leu-His and grown at 30°C for 2–3 d.

### Western blotting

Total protein extracts were prepared via TCA precipitation and dissolved in 1× SDS buffer (50 mM tris-Cl, pH 8, 2% [wt/vol] sodium dodecyl sulfate, 0.1% [wt/vol] bromophenol blue, 10% [vol/vol] glycerol, and 100 mM β-mercaptoethanol; Grallert and Hagan, 2017a). The proteins were separated on either 10% acrylamide or NuPAGE 4–12% Bis-tris (NP0322; Thermo Fisher Scientific) gels. Working concentrations of affinity-purified anti-Sid4 were ∼1:1,000. Tissue culture supernatant TAT1 was used at a dilution of 1:500, cdc2 antibody (Ab5467; Abcam) was 1:1,000, and in-house antibodies raised to and affinity purified with recombinant GFP were used at 1:1,000. Alkaline-phosphatase–coupled secondary antibodies (Sigma-Aldrich) were used for all blots, followed by direct detection with NBT/BCIP substrates on nitrocellulose membranes.

Detection with Sid4 antibodies that recognize Sid4 when phosphorylated on T584 and simultaneously on both T275 and S278 were performed on immunopurified Sid4 from TCA-precipitated whole-cell extracts. 2 × 10^8^ cells were used for each immunoprecipitation (Grallert and Hagan, 2017b). The cells were lysed in the presence of 200 µl of 20% TCA and 0.5 ml ice-cold acid washed glass beads using a Yasui Kikai multibead shocker at 4**°**C (2,500 rpm for 30 s in the beater, which had been precooled to 4°C). The precipitated proteins were pelleted by centrifugation at 14,000 *g* at 4°C and washed 2 × 1 ml with 0.1% ice-cold TCA. The pelleted protein was subsequently dissolved in 100 µl of immunoprecipitation buffer (50 mM tris-Cl, pH 8, 50 mM NaCl, 1 mM EDTA, 20 mM Na-β-glycerophosphate, 0.1 mM Na_3_VO_4_, 1 mM DTT, 0.2% triton-X100, protease inhibitor cocktail [11836153001; Roche], PhosSTOP [4906845001; Roche], and 1 mM PMSF) containing 2% SDS and heated to 80°C for 10 min. The denatured protein suspension was diluted further with 1 ml of immunoprecipitation buffer + 1% triton X-100 and centrifuged at 14,000 *g* at 4°C to clear the denatured protein cell lysate. Denatured Sid4 was immunopurified using Sid4 antibodies and Protein A/G Magnetic Beads (88803; Thermo Fisher Scientific). The primary antibody was diluted 1:20 in blocking buffer 1 (1% BSA, 100 mM lysine HCl, 10 nM NaH_2_PO_4_, 50 mM tris-Cl, pH 7.5, 150 mM NaCl, and 0.05% Tween-20), and the alkaline-phosphatase–coupled secondary rabbit antibody (A3687; Sigma-Aldrich) was diluted 1:10,000 and probed in blocking buffer 2 (1% BSA, 100 mM lysine HCl, 50 mM tris-Cl, pH 7.5, 150 mM NaCl, and 0.05% Tween-20). Blots were detected with NBT/BCIP substrates on PVDF membranes.

### Coimmunoprecipitation of recombinant GFPn.Sid4(466–660) fragments and Ppc89.3Pk

Expression of N-terminally GFP-tagged *sid4* fragments (aa 466–660; Fig. 2 A) was induced by culture in thiamine-free medium at mid-log phase for 24 h at 25°C to a final cell density of 4 × 10^6^ cells/ml. Cells were subsequently harvested via centrifugation at 5,500 *g* for 1 min and washed once with ice-cold STOP buffer (10 mM EDTA, 50 mM NaF, 150 mM NaCl, and 1 mM NaN_3_; Simanis and Nurse, 1986), centrifuged at 11,000 *g* for 1 min at room temperature to remove the supernatant, and snap-frozen in liquid N_2_ for storage at –80°C. For 4 × 10^8^ cells, 200 µl of IP1 buffer (50 mM Tris-Cl, pH 8, 50 mM NaCl, 1 mM EDTA, 20 mM Na-β-glycerophosphate, 0.1 mM Na_3_VO_4_, 1 mM DTT, 100 mM lysine HCl, 0.5% triton-X100, protease inhibitor cocktail, and 1 mM PMSF) was used (Grallert and Hagan, 2017b). The GFP-tagged Sid4 fragments were precipitated using GFP-Trap–M (Gtm-20; ChromoTek), and coprecipitation of Ppc89.3Pk was assayed by Western blotting with monoclonal antibodies against the SV5 Pk epitope (MCA1360; Bio-Rad Laboratories) at a 1:1,000 dilution from stock.

### Coimmunoprecipitation of Sid4 and Chk2^Cds1^.GFP

The approach of LaCava et al. (2016) was used to identify conditions for coprecipitation. Cells were grown in EMM2 at 25°C to a cell density of 4 × 10^6^ cells/ml and subsequently harvested via centrifugation at 5,500 *g* for 1 min and washed once with ice-cold STOP buffer (10 mM EDTA, 50 mM NaF, 150 mM NaCl, 1 mM NaN_3_, and 1 mM PMSF)^16^. After further centrifugation at 5,500 *g* for 1 min, the supernatant was removed, and the cell paste was passed through a 10-ml syringe into liquid N_2_ to form frozen yeast cell “noodles.” The frozen cells were subsequently ground in a 6870 Freezer/Mill (SPEX SamplePrep) in 10 × 2–min grinding cycles set at level 10 with 2-min breaks at each interval. The ground cell powder was weighed and aliquoted into screw-capped tubes that were precooled by immersion in liquid N_2_. 4 × 100 mg of ground cells were resuspended in 400 µl of IP2 buffer (50 mM Hepes, pH 7.5, 150 mM NaCl, 50 mM NaF, 60 mM Na-β-glycerophosphate, 0.2 mM Na_3_VO_4_, 10% glycerol, 1 mM DTT, 2 mM EDTA, 0.2% triton X-100, protease inhibitor cocktail, and 1 mM PMSF). The resuspended cell lysate was centrifuged at 14,000 *g* at 4°C for 5 min, and the cleared lysate was pooled into a single tube. Chk2^Cds1^.GFP was precipitated using GFP-Trap–MA (Gtma-20; ChromoTek), and coprecipitation of Sid4 was assayed by Western blotting.

### Protein kinase assays

Assessment of the activities of NIMA^Fin1^, Sid2, and Pom1 toward recombinant Sid4 were conducted as reported previously, with the exception that recombinant Sid4 (see the Antibody generation section) was substituted for the previously described substrates (Bähler and Nurse, 2001; Grallert et al., 2012). NIMA^Fin1^/Sid2 was precipitated from 2 × 10^8^ cells with polyclonal antibodies with DynaBeads A (Invitrogen) in KA buffer (50 mM Hepes, 10 mM EDTA, 40 mM Na-β-glycerophosphate, 4 mM Na_3_VO_4_, 50 mM NaF, 0.6% NP-40, 150 mM NaCl, protease inhibitor cocktail, and 1 mM PMSF). The kinase reaction was performed at 30°C for 30 min in KR buffer (20 mM Hepes, 15 mM KCl, 1 mM EGTA, 10 mM MgCl_2_, 10 mM MnCl_2_, 0.125 nM ATP, and 10 µg of recombinant Sid4). For Pom1 assays, 1.5 × 10^8^ cells were harvested by filtration, washed in STOP buffer, and once in POM buffer (2.5 mM Hepes, pH 7.4, 1% triton X-100, 10% glycerol, 50 mM KCH_3_CO_2_, 50 mM NaF, 60 mM Na-β-glycerolphosphate, 2 mM EDTA, 1 mM dithiothreitol, 0.1 mM Na_3_VO_4_, 15 mM *p*-nitrophenylphosphate, protease inhibitor cocktail, 1 mM PMSF, and 2 mM benzamidine) before resuspension in POM buffer. After disruption by agitation with glass beads in a Yasui Kikai multibead shocker (2,500 rpm for 30 s in the beater, which had been precooled to 4°C) and clearing of the cell supernatant, 9-E10 monoclonal antibodies that recognize the myc epitope were added along with DynaBeads A (Invitrogen) before incubation for 2 h with agitation at 4°C. After two washes in POM buffer and one in AB buffer (25 mM MOPS, pH 7.0, 60 mM Na-β-glycerolphosphate, 7 mM MgCl_2_, 7 mM MnCl, 0.1 mM Na_3_VO_4_, protease inhibitor cocktail, and 2 mM benzamidine), the beads were resuspended in AB buffer plus 10 µg of recombinant Sid4 and 10 mM ATP and then incubated at 30°C for 20 min. Control experiments with the validated substrates ensured that the conditions to assay activity had been established (not depicted).

### Mass spectrometry

Sid4 protein was isolated from *cdc2.3w wee1.50* cells (cells undergoing mitotic catastrophe; Russell and Nurse, 1987) by denaturing immunoprecipitation according to Grallert and Hagan (2017c). 4 × 10^10^ cells were isolated by centrifugation from mid-log phase cultures that had been grown overnight at 25°C before incubation at 36°C for 90 min. After washing in STOP buffer, cells were resuspended in 2 ml ice-cold 20% TCA before snap freezing in liquid nitrogen to produce yeast “noodles.” After resuspension of the powder generated by cell disruption in a SamplePrep 6870 freezer mill (SPEX) in 40 ml ice-cold 5% TCA and centrifugation at 20,000 *g* for 10 min at 4°C, the supernatant was discarded before a further wash with 0.1% TCA. The pellet was resuspended in 10 ml immunoprecipitation buffer (100 mM tris, pH 8, 50 mM NaCl, 1 mM EDTA, 20 mM Na-β-glycerolphosphate, 0.1 mM Na_3_VO_4_, 50 mM NaF, 0.2% SDS, 2 mM PMSF, and protease inhibitor cocktail) containing 4% SDS. After 3 min at 70°C, 90 ml of ice-cold immunoprecipitation buffer containing 1% triton X-100 was added before centrifugation at 20,000 *g* for 10 min at 4°C. Denatured Sid4 was immunopurified from this supernatant using Sid4 antibodies chemically conjugated to Protein A/G Magnetic Beads. Isolated Sid4 was subsequently analyzed via mass spectrometric analysis according to Unwin et al. (2005). Samples were run on 4–12% NuPAGE bis-tris gel (Invitrogen), and Sid4 bands were excised and digested with either 20 ng sequencing-grade trypsin (Sigma-Aldrich), 400 ng LysN (Associates of Cape Cod), or 350 ng elastase (EMD Millipore) in 100 µl 40 mM ammonium bicarbonate with 9% (vol/vol) acetonitrile at 37°C for 18 h. The peptides were separated using a nano-acquity UPLC system using a nano-acquity BEH C18 column (75 µm inner diameter, 1.7 µm, 25 cm; Waters) with a gradient of 1–25% (vol/vol) of acetonitrile with 0.1% formic acid over 30 min at a flow rate of 400 nl/min. The LTQ-Orbitrap XL mass spectrometer was operated in parallel data-dependent mode where the mass spectrometry survey scan was performed at a nominal resolution of 60,000 (at m/z 400) in the Orbitrap analyzer over an m/z range of 400–2,000. The top six precursors were selected for collision-induced dissociation in the LTQ at a normalized collision energy of 35% using multistage activation at m/z 98.0, 49.0, and 32.7 D.

### Online supplemental material

Fig. S1 shows how *sid4.soc* but not *sid4.T584V* mutations can suppress the SPB activation defect of *cut12.1*. Fig. S2 shows how anchorage of Sid4.T584EGFP to SPB-associated Ppc89 suppresses the temperature sensitivity arising from the T584E phosphomimetic mutation, whereas anchorage of CK1δ^Hhp2^ to Sid4 SPB or phosphomimetic mutation of T275S278 suppresses the SPB activation defect of *cut12.1*. Fig. S3 shows how suppression of *cut12.1* by mutation of *sid4* relies upon Chk2^Cds1^ recruitment. Fig. S4 shows the eviction of Flp1.GFP from the SPBs of *sid4*^+^ and *sid4.T275ES278D* upon HU treatment. Table S1 lists the strains used in the study.
